# Knowledge, attitude and practice of physiotherapists towards health promotion in Ghana

**DOI:** 10.4102/sajp.v74i1.443

**Published:** 2018-08-28

**Authors:** Hosea Boakye, Jonathan Quartey, Nana A.B. Baidoo, Josephine Ahenkorah

**Affiliations:** 1 Department of Physiotherapy, LEKMA Hospital, Ghana; 2 Department of Physiotherapy, University of Ghana, Ghana; 3 Lifebridge Physiotherapy Clinic, Accra, Ghana

## Abstract

**Background:**

Physiotherapists are well equipped to address health promotion issues with their patients and the public. However, no studies have been conducted in Ghana to assess the knowledge, attitude and practice of physiotherapists towards health promotion.

**Objectives:**

This study therefore seeks to determine the knowledge, attitude and practice of physiotherapists towards health promotion in Ghana.

**Methods:**

This cross-sectional study was conducted at some selected physiotherapy departments in health facilities across Ghana. Ninety-one registered physiotherapists living and working in Ghana were recruited for this study. A closed-ended self-administered questionnaire was used to collect data on the demographics, knowledge, attitude and practice of physiotherapists towards health promotion. The scores for each section were calculated individually, and the final knowledge, attitude and practices score was obtained by calculating the total of the three sections. Statistical Package for Social Sciences version 22.0 was employed to analyse all the study variables.

**Results:**

Physiotherapists’ knowledge was 72%, attitude 84% and practice 87% towards health promotion. The association between the physiotherapists’ knowledge of health promotion and practice was significant with Pearson’s chi-square test (*p* = 0.013). But there was no significant association between knowledge and attitude of physiotherapists towards health promotion (*p* = 0.097).

**Conclusion:**

Physiotherapists have very good knowledge, attitude and practice towards health promotion in Ghana. This is essential for better integration into the scope of physiotherapy practice, and therefore, the health promotion policy in Ghana should be revised to include physiotherapists, because they are experts in exercise prescription and physical activity.

**Clinical implications:**

The outcomes of this study could provide the impetus for physiotherapists to include health promotion in clinical and community services for primary prevention of non-communicable diseases as well as secondary and tertiary prevention of disability to promote functional independence.

## Introduction

Health promotion, according to the Ottawa Charter, is ‘the process of enabling people to increase control over and to improve their health’ (WHO 1986). In a broader interpretation, the Ghana National Health Promotion Policy (2005) states that health promotion concerns all the experiences of an individual, group or community that influence beliefs, attitudes and behaviour with respect to health as well as the process and the efforts of producing changes when this is necessary for optimum health. The Ghana National Health Promotion Policy (2005) further explained that health promotion is a means of increasing individual and collective participation in health action and strengthening programmes through the integrative use of documented guidelines. To strengthen the health promotion policies in achieving the United Nations Development Agenda 2030 and its sustainable development goals (SDGs), the Shanghai declaration on promoting health in the 2030 Agenda for Sustainable Development advocates the implementation of the SDGs through increased political commitment and financial investment in health promotion (WHO 2017).

Physiotherapists are primary health care professionals who use approaches that intend to promote, maintain and restore physical, psychological and social well-being (Perreault 2008; Shirley, Van-der Ploeg & Bauman 2010). The biomedical model states that health constitutes freedom from disease, pain or defect, thus making the normal human condition healthy (Perreault 2008). The biomedical model, according to Annandale (1998), focuses on physical aspects such as pathology, biochemistry and the physiology of a disease, but does not consider the role of psychological, environmental and social influences. Empowerment, according to Perreault (2008), which is a central concept in health promotion, is probably not facilitated in physiotherapy interventions based on the biomedical model, hence the introduction of the bio-psychosocial model. This model according to Havelka, Lucanin and Lucanin (2009) takes into account the biological, psychological and social factors in the assessment, prevention and treatment of diseases. Therefore, knowledge of physiotherapists about health promotion will guide them to include all relevant measures (biomedical, social and psychological) in their practice.

Nyamwaya (2003) stated that Africa has made a delayed entry into the world of health promotion, and there is not much published work about the role of health promotion among physiotherapists in Africa. It seems that very little research has addressed physiotherapy and health promotion in sub-Saharan Africa.

Ghana, like other sub-Saharan African countries, is experiencing a double burden of disease. Communicable diseases are prevalent, while non-communicable diseases are gradually increasing (Ghana National Health Promotion Policy 2005). Non-communicable diseases, especially cardiovascular diseases, represent the major health burden in industrialised countries and are a rapidly growing problem in developing countries. A well-planned and determined community-based health promotion programme can curb the alarming rate of this condition when it reflects on the lifestyle of individuals (Pekka 2002). Furthermore, one of the challenges the Health Promotion Unit of Ghana Health Service has, is a lack of adequately trained personnel, at all levels, to embark on effective and sufficient health promotion activities. Currently, professionals recognised by the Ghana Health Service involved in health promotion and public health are medical doctors, nurses, health educators and promoters with no physiotherapists on board.

The World Confederation for Physical Therapists (WCPT) (2007) advocates for the integration of health promotion in physiotherapy practice or in the curricula (training). Physiotherapists are therefore in a good position to address health promotion issues with their patients and the public (Healey et al. 2012; Taukobong et al. 2014). Yet, little is known about the actual health promotion practice or the confidence of physiotherapists to engage in such activities and the benefits of doing so (Rea et al. 2004).

Rea et al. (2004) reported that some health promotion practices in physiotherapy include educating persons with disabilities on healthy ways of living to prevent secondary conditions like obesity and hypertension; educating the public on physical fitness and activity; and informing the public of the dangers of tobacco use and education on healthy living of people with arthritis, osteoporosis, chronic low back pain, heart disease and stroke. Joseph (2011) also enumerated some health promotion practices in physiotherapy which include physiotherapists being part of road safety campaign organisations, which emphasise the necessity of drivers’ and passengers’ use of seat belts and the use of car seats for children. This is helpful in reducing the number of serious injuries that may occur. Furthermore, physiotherapists can participate in school programmes to provide educational information regarding the importance of physical exercise and the risk of obesity in pupils and students (Joseph 2011). This implies that the role of physiotherapy towards health promotion does not end in the clinic but extends to the public as well.

Another way to facilitate effective health promotion practices within the scope of physiotherapy is for physiotherapists to attend continuing professional development (CPD) programmes. Although there is empirical evidence about the significant contribution of physiotherapists in health promotion, there appears to be a dearth of published work about the role of physiotherapists in Ghana about health promotion.

This view adds credence to this study, the aim of which was to ascertain the knowledge, attitude and practices (KAPs) of physiotherapists towards health promotion in Ghana.

## Methodology

This cross-sectional study was conducted at nine different physiotherapy departments across Ghana, consisting of three teaching hospitals, four regional hospitals and two district hospitals. These hospitals were purposively selected based on the number of physiotherapists working there.

Ninety-one physiotherapists, registered with the Ghana Physiotherapy Association, consented to take part in the study.

### Instrument for data collection

The questionnaire used in this study was adapted from a previous study by Joseph (2011). The content validity of the questionnaire was carried out to suit the local context of this study by two physiotherapists (lecturers) from the University of Ghana. The lecturers peer-reviewed the questionnaires, and a few recommendations were made; a reliability test, by means of a test–retest procedure 2 weeks apart, was performed among 10 physiotherapists, to ascertain the clarity of the questionnaire. The test–re-test procedure produced a reliability of 0.72.

This closed-ended questionnaire has four sections. Section A described the demographic data of the participants. Section B had questions on health promotion practice. Section C contained questions on the attitude of physiotherapists towards health promotion, while Section D had questions about the physiotherapist’s knowledge of health promotion.

Questions in sections B, C and D tested the KAP of the participants. The scores for each section were calculated individually, namely knowledge (Section D), attitude (Section C) and practice (Section B); and the final KAP score was obtained by calculating the total of the three sections. The scores were categorised as 80% – 100% (very good), 60% – 79% (good), 50% – 59% (fair) and 0% – 49% (unsatisfactory).

### Procedure for data collection

Information with regard to the study and dates that the authors would visit the various hospitals was sent to all the physiotherapists in the selected hospitals via their email addresses. A text message reminding the physiotherapists of the date the authors would visit their departments was sent 2 days prior to the visit.

One of the authors visited all the selected hospitals at the agreed date and distributed the questionnaires. A written informed consent form explaining the rationale of the study accompanied the questionnaire. Once the physiotherapists consented to participate in the study, they completed the questionnaires, which were retrieved on the same day.

### Data analysis

The data obtained were entered into a database and analysed using the Statistical Package for Social Sciences (SPSS) version 20. Descriptive statistics of frequency distributions, pie charts and percentages were used to represent the data obtained. Chi-square analysis was used to test for association between knowledge and attitude as well as knowledge and practice of physiotherapists towards health promotion.

### Ethical considerations

Ethical approval was sought and obtained from the Ethics and Protocol Review Committee of the School of Allied Health Sciences, University of Ghana (SAHS-ET./10308759/AA/6A).

## Results

The authors were able to reach 91 out of a possible 131 registered physiotherapists in the selected hospitals (excluding those who participated in the reliability testing). All 91 questionnaires were retrieved accounting for a 100% response rate. Some physiotherapists could not be recruited as some were abroad for studies and others were on annual leave during the time of data collection.

### Demographic characteristics of respondents

**FIGURE 1:**
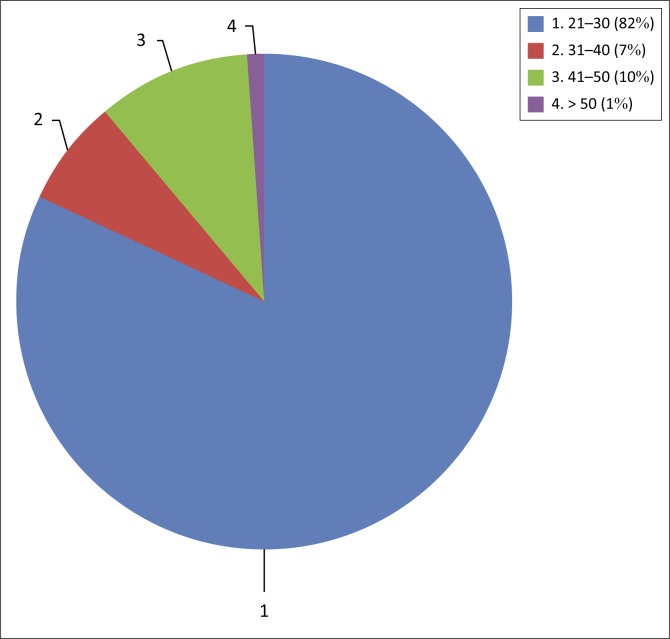
Pie chart showing the age distribution of participants.

**FIGURE 2:**
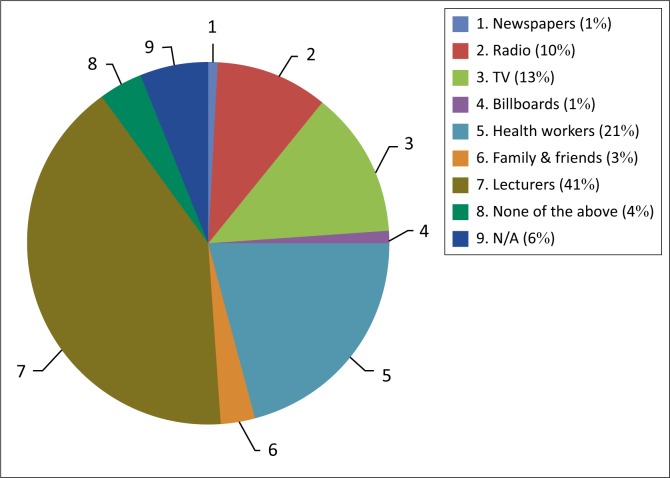
Pie chart showing where physiotherapists were first exposed to health promotion.

The results show that most of the participants 75 (82%) were from the age group 21–30 years as shown in Figure 1. Out of the 91 respondents, 48 (53%) were female. The results indicate that 37 (41%) of the respondents knew or had heard about health promotion from their lecturers (Figure 2). The majority (81 respondents, 89%) of the participants had qualified from the University of Ghana, while the remaining 10 (11%) qualified from universities abroad.

### Health promotion coverage and exposure

**FIGURE 3:**
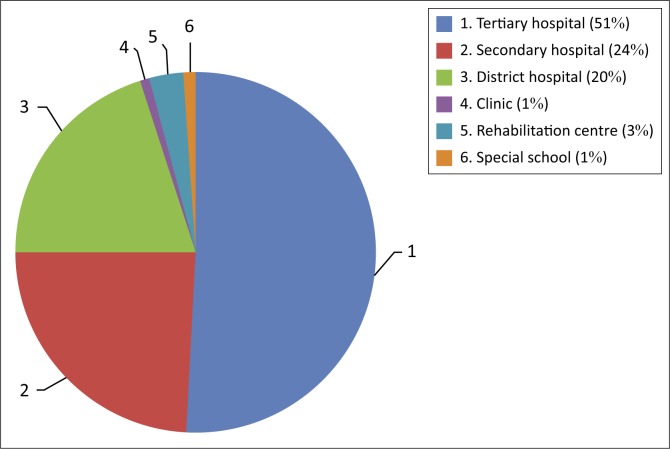
Pie chart showing institutions where physiotherapists are currently working.

Half of the respondents (46 respondents, 51%) currently worked in a tertiary or academic hospital, as shown in Figure 3. Most of the respondents (76 respondents, 83.5%) had 0–5 years working experience, 10 (11.0%) had 6–10 years working experience, 3 (3.3%) had 11–15 years working experience and 2 (2.2%) had more than 20 years working experience. Almost all of the respondents (89 respondents, 98%) were familiar with the concept of health promotion, while 2 (2%) respondents were not.

The results showed that 85 (93%) of the respondents had covered health promotion during their undergraduate training, while 6 (7%) respondents had not. The results also show that only 25 (27%) of the respondents had informal training in health promotion from workshops after obtaining undergraduate degrees, while 66 (73%) respondents had none.

### Practices towards health promotion

The results show that 88 (96.7%) of the respondents practise health promotion at their workplaces by advising others to make use of a seat belt when driving to prevent serious injuries and providing appropriate ergonomic workstation arrangements at workplaces, and they educated clients on correct posture and methods of lifting heavy objects as shown in Table 1.

**TABLE 1:** Physiotherapy practice on health promotion.

No.	Question	Yes *n* (%)	No *n* (%)
1	Are you practising health promotion at your work as a physiotherapist?	88(96.7)	3(3.3)
2	Do you advise others to make use of seat belt to prevent serious injuries?	73(80.2)	18(19.8)
3	Are you involved in providing ergonomically appropriate work environment in your workplace?	71(78.0)	20(22.0)
4	Are you educating your clients on correct posture and method of lifting heavy objects?	91(100.0)	0(0.0)
5	Are you educating your clients on not using sweetened beverages in order to prevent diabetes?	61(67.0)	30(33.0)
6	Physiotherapy services include planning, organisation and evaluation of health promotion activities.	84(92.3)	7(7.7)
7	Physiotherapy intervention includes training of patients, caregivers and family to apply preventive, curative and promotive measures.	88(96.7)	3(3.3)
8	Exercises are the only means of promoting health in all conditions treated.	2(2.2)	89(97.8)

*n*, is number of respondents.

Percentage is presented in brackets.

### Attitude towards health promotion

Twenty-eight (30.8%) participants rated their attitude towards health promotion as excellent as shown in Table 2. All the 91 (100%) participants showed a positive attitude towards health promotion by indicating that they would educate their clients on healthy diet and the need for exercise. Some participants 29 (31.9%) indicated that there is no need for physiotherapy to shift from the biomedical approach to a model ensuring health promotion as shown in Table 3.

**TABLE 2:** Self-reported attitudes towards health promotion.

Practice	Total *n* (%)
Excellent	28(30.8)
Good	61(67.0)
Poor	1(1.1)
N/A	1(1.1)

*n*, is number of respondents.

Percentage is presented in brackets.

**TABLE 3:** Physiotherapists’ attitude to health promotion.

No.	Question	Yes *n* (%)	No *n* (%)
1	Would you get involved in the efforts to improve physical exercise for school children to reduce the prevalence of obesity and related diseases?	84(92.3)	7(7.7)
2	Would you participate in an advocacy activity to electrify households to reduce burns by the use of coal and paraffin?	72(79.1)	19(20.9)
3	Would you educate all your clients on healthy diet and need for exercise?	91(100)	0(0.0)
4	Would you play a part in the ‘arrive alive’ campaign of the government?	83(91.2)	8(8.8)
5	Would you educate people on ergonomics in the community where you practise?	88(96.7)	3(3.3)
6	Would you partake in developing health and safety regulation to prevent silicosis?	79(86.8)	12(13.2)
7	The most appropriate role for physiotherapist is that of a health educator.	52(57.1)	39(42.9)
8	There is a need for physiotherapy to shift from a biomedical approach to a model ensuring health promotion.	62(68.1)	29(31.9)
9	Physiotherapy should only concern itself with curing the symptom in a patient.	14(15.4)	77(84.6)
10	Physiotherapists working within the district health system should include health promotion in their services.	89(97.8)	2(2.2)
11	Physiotherapists working within the district health system should include health promotion in their services.	12(13.2)	79(86.8)

*n*, is number of respondents.

Percentage is presented in brackets.

### Knowledge towards health promotion

The majority of participants 90 (98.9%) indicated that disease prevention programmes, such as vaccination, is a method of health promotion. Other results are shown in Table 4.

**TABLE 4:** Physiotherapists’ knowledge of health promotion.

No.	Statement	Yes *n* (%)	No *n* (%)
1	Health promotion activity involves building healthy public policy to promote health of the population.	91(100.0)	0(0.0)
2	Health promotion activity involves distribution of prophylactic medication to prevent disease.	60(65.9)	31(34.1)
3	Health promotion activity involves early detection and treatment of disease.	74(81.3)	17(18.7)
4	Health promotion activity involves strengthening community action to prevent disease and improve health.	88(96.7)	3(3.3)
5	Health promotion activity involves developing personal skill to stay healthy.	82(90.1)	9(9.9)
6	Health promotion is implemented by treating the diseases of people in the community.	43(47.3)	48(52.7)
7	Health education is a process of implementing health promotion.	82(90.1)	9(9.9)
8	Health promotion can be achieved through environmental modification.	83(91.2)	8(8.8)
9	Disease prevention programmes such as vaccination is a method of health promotion.	90(98.9)	1(1.1)
10	Health promotion includes the implementation of lifestyle and behavioural change programme.	88(96.7)	3(3.3)
11	Provision of basic services such as housing, clean water, sanitation and adequate nutrition is part of a health promotion programme.	86(94.5)	5(5.5)
12	Health promotion calls for re-orientation of health services beyond the clinical and curative services.	89(97.8)	2(2.2)

*n*, is number of respondents.

Percentage is presented in brackets.

### Knowledge, attitude and practice towards health promotion

According to the score chart, there was an overall desirable (80% – 100%) score of 81%, of all physiotherapists’ KAP. These are depicted in Table 5.

**TABLE 5:** Knowledge, attitude and practice scores chart.

Category	Score chart (%)
Knowledge	72
Attitude	84
Practice	87
Average	81

### Associations between knowledge and attitude and knowledge and practice

The chi-square (*c*^2^) and *p*-values were calculated at degrees of freedom (*df*) as shown in Tables 6 and 7.

**TABLE 6:** Association between knowledge and attitude.

Knowledge	Attitude			
	Unsatisfactory	Fair	Good	Desirable
Unsatisfactory	0	0	1	0
Fair	0	1	2	3
Good	2	0	13	54
Desirable	0	1	2	12

Note: *df* = 9; *c*^2^ = 14.785; *p* = 0.097.

**TABLE 7:** Association between knowledge and practice.

Knowledge	Practice		
	Fair	Good	Desirable
Unsatisfactory	0	0	1
Fair	1	2	2
Good	0	14	55
Desirable	0	2	13

Note: *df* = 6; *c*^2^ = 16.055; *p* = 0.013*.

* , significant at *p* ≤ 0.05.

## Discussion

The KAPs of physiotherapists towards health promotion in Ghana are essential, if better integration into the scope of physiotherapy practice is to be promoted.

It is encouraging that the majority of the participants reported that health promotion, which is part of physiotherapy, was covered during their undergraduate training. In other words, health promotion was adequately catered for in their undergraduate curricula. Joseph (2011) also reported that the majority of physiotherapists trained in South Africa covered health promotion at an undergraduate level.

Some studies have advocated for the integration of disease prevention and health promotion into undergraduate physiotherapy curricula. This may be useful to build students’ confidence on how to apply skills and knowledge in disease prevention and health promotion, also preparing them for practice as physiotherapists.

As health promotion is catered for in undergraduate physiotherapy curricula, it is expected that the KAP of physiotherapists towards health promotion would increase with time depending on the level of attention (CPD programmes) given to health promotion during practice. The few respondents who reported having had additional training in health promotion from workshops could be attributed to the low turnout of physiotherapists at CPD programmes in health promotion or that very few CPD programmes about health promotion were organised by the professional body. It therefore behoves the Ghana Physiotherapy Association to organise more CPD programmes that focus on or emphasise health promotion for physiotherapists to be abreast with current trends of health promotion practices in physiotherapy, which may invariably influence their practice.

### Knowledge towards health promotion

Although a lack of knowledge has been reported to be a possible barrier to practice, our study shows that physiotherapists have good knowledge of health promotion, which could be an opportunity for physiotherapists working in Ghana to integrate health promotion into their scope of practice.

Although the majority of the respondents covered health promotion during their undergraduate training, this study identified some deficiencies about their knowledge as most respondents indicated that health promotion activities involve early detection and treatment of disease. Additionally, the majority of the participants responded that health promotion involves the distribution of prophylactic medication to prevent disease. The result of this study appears to indicate that there are some physiotherapists who do not understand how health promotion differs from a biomedical perspective and this could probably affect application in practice.

This according to Joseph (2011) is an indication that physiotherapists still require further training about implementing and understanding their role in health promotion. As a result, adequate CPD workshops in health promotion should be organised for physiotherapists to broaden their knowledge.

### Practice towards health promotion

Some studies (Healey et al. 2012; Mulligan et al. 2012; Shirley et al. 2010; Taukobong et al. 2014) report that physiotherapists are well positioned to practise health promotion interventions and that it should form an integral part of physiotherapy. The majority of physiotherapists who participated in this study seem to engage in health promotion practices at their workplace, by offering education about the prevention of diabetes and also indicated that their knowledge of exercise prescription is not the only means of health promotion.

The positive responses also show that the application of ergonomic principles is crucial in health promotion (Shirley et al. 2010). Ergonomics is concerned with the promotion of health, productivity, safety and comfort of an individual and is practised among physiotherapists to restore normal functional activities. Physical activity is also accepted worldwide as a public health priority (Shirley et al. 2010) and physiotherapists have more extensive training on exercise prescription for both the primary and tertiary prevention of diseases and disability (Aweto et al. 2013) than other health care professionals (Perreault 2008).

Therefore, the Ministry of Health in Ghana is encouraged to make room for physiotherapists to be part of the government’s health promotion policies and to be part of the health promotion personnel who are involved in the community for health promotion programmes or activities.

### Attitude towards health promotion

The physiotherapists’ perceived high positive responses about their attitude towards health promotion could be considered a good sign for the scope of physiotherapy practice and health promotion in Ghana. This perceived positive attitude towards health promotion was confirmed by the majority of physiotherapists’ responses that they educate people on ergonomics in the community where they practise.

Although all respondents indicated that they would educate their clients on a healthy diet and the need for exercise, there is still a need for physiotherapists to shift from a biomedical approach to a model that ensures health promotion. In line herewith, almost a third of the respondents indicated that there is no need for physiotherapy to shift from a biomedical approach to a model that will ensure health promotion. Most practising physiotherapists are probably not aware of the distinctions among the different models of health as is also reported by Joseph (2011). Therefore, further education and training should be given to address this issue.

Most of the respondents felt that the most appropriate role for physiotherapist is that of a health educator which is not different from reports by Perreault (2008), which indicated that health education is the most used health promotion strategy in physiotherapy practice.

### Knowledge, attitude and practice towards health promotion

As the average and overall attitude and practice of the physiotherapists were desirable and knowledge was good, a comparison of the overall KAP scores of physiotherapists in Ghana appears higher than that of their counterparts in the Gauteng province of South Africa (Joseph 2011). This could be because of the high number of physiotherapists working in Ghana who covered health promotion during their undergraduate training as compared to those working in South Africa. It could therefore be said that physiotherapists working in Ghana were better exposed to the principles of health promotion, which possibly led to a very good KAP in health promotion.

### Association between knowledge and practice

The association between knowledge and practice of physiotherapists towards health promotion buttresses the need for more CPD workshops for physiotherapists to practise health promotion appropriately. This could mean the higher the knowledge of a physiotherapist in health promotion, the more appropriate the practice thereof.

### Association between knowledge and attitude

The knowledge of participants did not seem to influence their attitude towards health promotion, as there was no association between knowledge and attitude towards health promotion. It could therefore be said that participants may have low knowledge about health promotion but still have a positive attitude towards health promotion and vice versa. Therefore, the level of knowledge of health promotion does not necessarily determine an individual’s attitude towards health promotion.

## Conclusion

Physiotherapists in Ghana have good KAP towards health promotion. This is essential for better integration into the scope of physiotherapy practice and could support the inclusion of physiotherapists in the health promotion policy in Ghana to further enhance health promotion in the country.
